# Evaluating the stability of antibody titres against *Leishmania infantum* determined by IFAT in long-term stored canine frozen samples

**DOI:** 10.1186/s13071-025-06982-x

**Published:** 2025-08-03

**Authors:** Patricia Olmeda, David Díaz-Regañón, Alejandra Villaescusa, Inmaculada Amusategui, Miguel A. Tesouro, Fernando Rodríguez-Franco, Mercedes García-Sancho, Daniel Martín-Fraile, Ángel Sainz

**Affiliations:** 1 Department of Animal Medicine and Surgery, College of Veterinary Medicine, Complutense University of Madrid, Avda. Puerta de Hierro S/N, 28040 Madrid, Spain; 2 Department of Veterinary Medicine, Surgery and Anatomy, College of Veterinary Medicine, University of León, Campus de Vegazana, 24071 León, Spain

**Keywords:** Leishmaniosis, Diagnosis, Biological preservation, Plasma, Dogs

## Abstract

**Background:**

The immunofluorescence antibody test (IFAT) is a serological diagnostic technique used to quantify serum antibodies generated in response to exposure to various pathogens, such as *Leishmania infantum*. Retrospective analysis of previously collected frozen samples is highly valuable for clinical and research purposes. The primary aim of this study was to evaluate the impact of long-term frozen storage of canine plasma samples on the IFAT-based serological diagnosis of *L. infantum* exposure.

**Methods:**

A total of 189 frozen plasma samples from dogs stored at −20 °C for 5, 10 or 20 years, which had previously been tested for *L. infantum* exposure via IFAT (IgG), were reanalysed to assess the concordance between past and current qualitative and quantitative results.

**Results:**

The qualitative agreement between the former and current IFATs was 92.1%. The samples from 20 years prior presented the greatest increase in negative samples in the second analysis (from 28.6 to 39.7%). A strong positive correlation was observed between the quantitative measurements of the past and present across all three groups. The exact quantitative agreement was 48.7%.

**Conclusions:**

The results of this study indicate that freezing at −20 °C is a good technique for prolonged storage of samples for the detection of *L. infantum* exposure in dogs, as the qualitative IFAT result is not significantly altered. This finding is of particular interest both for clinical endeavours and for future research in this field.

**Graphical Abstract:**

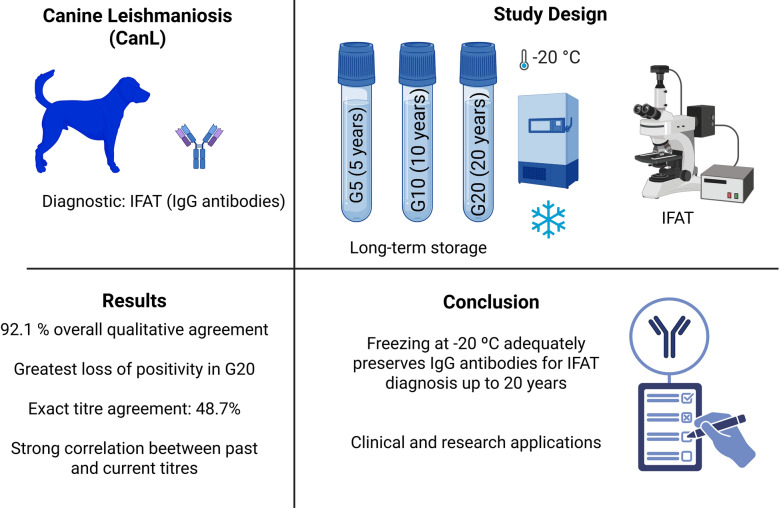

## Background

The protozoan *Leishmania infantum* is a causative agent of leishmaniosis, in which canines act as the primary reservoir host. The principal method of transmission is via the bite of a female phlebotomine sandfly [1–3]. Infection with *L. infantum* in dogs can have a broad range of outcomes, and the development of canine leishmaniosis (CanL) is not always a mandatory consequence. Furthermore, CanL is an insidious and pleomorphic disease that can manifest with a multitude of clinical signs and a wide range of severity grades, ranging from a self-limiting disease to a fatal illness [1].

The detection of immunoglobulin G (IgG) against *L. infantum* by quantitative serology represents the technique of choice for the diagnosis of CanL, although it can be complemented by other techniques [1, 4–7]. The immunofluorescence antibody test (IFAT) is a serological technique that quantifies the level of antibodies produced by a dog in response to *L. infantum* exposure [1, 8, 9]. This quantification is highly important, as dogs presenting clinical signs and clinicopathological alterations, along with elevated antibody levels defined as a 3–4-fold increase above the cut-off level, are generally confirmed to have CanL. Notably, the presence of low levels (1–2-fold elevation above the cut-off level) of IgG does not necessarily indicate the presence of disease, and further investigation is needed in such cases [1, 2, 6–11].

The preservation and subsequent analysis of serum or plasma samples may be valuable for clinical application, as they allow for retrospective analysis of *L. infantum* exposure in freeze-preserved samples from dogs that were not initially collected for that purpose*.* It may also be useful in investigative contexts, as this preservation of the samples can be convenient for the study design of future research and for future studies with samples that were collected in advance. It is a standard practice to collect serum samples and store them at freezing temperatures until IFATs against *L. infantum* can be performed [12–18]. Despite the fact that the stability of various types of IgGs has been evaluated under different frozen storage conditions [19–27], to the best of the authors’ knowledge, no studies have been conducted to specifically assess the stability of anti-*L. infantum* IgG antibodies when used for IFATs and stored frozen for extended periods.

The objective of this study is to evaluate the concordance between the initial serological results and outcomes of a repeated IFAT on the same plasma samples that have been stored frozen for an extended period of 5, 10 and 20 years. It was hypothesised that the storage conditions would not significantly affect the reproducibility of the *L. infantum* IFAT results.

## Methods

### Study design

A total of 189 plasma samples that had previously been tested for detection of *L. infantum* exposure were re-evaluated. Samples from independent dogs were selected according to the year of their first serodiagnosis and divided into three groups (G5: 5 years ago; G10: 10 years ago; G20: 20 years ago). The study included 63 samples from each group. To ensure the broadest possible scope of analysis, a diverse array of titres was selected for inclusion from each timepoint (negative (<1/50), 1/50, 1/100, 1/200, 1/400, 1/800, 1/1600 and 1/6400). Samples from every antibody titre were randomly selected from among all the samples available in the plasma library. At the time of the initial diagnosis, the samples were stored at a temperature of −20 °C and maintained under these conditions until retesting for this study (July–November 2024). The plasma samples used in this study had not been previously employed in other experiments and had not undergone any prior freeze–thaw cycles.

### IFAT serodiagnosis

Plasma samples (25 μL) from canine blood were analysed at the Canine Leishmaniosis and Ehrlichiosis Diagnostic Service of the Complutense Veterinary Teaching Hospital of the Complutense University of Madrid. Serodiagnosis was performed by detecting specific antibodies against *L. infantum* using the indirect immunofluorescence antibody test for anti-*Leishmania*-specific IgG antibodies [28–31]. The antigen was obtained from a culture of promastigotes of *L. infantum* L-75 established in Novi, McNeal and Nicolle medium. In a microtiter plate of 96 wells, serial dilutions of the samples were made with phosphate-buffered saline (PBS): the first two wells had a 1/5 dilution, and the remaining wells had a 1/2 dilution. A positive control was also tested. Twenty microliters of the 1/50, 1/100, 1/400, 1/800, 1/1600 and 1/6400 dilutions were added to the wells of the *L. infantum* antigen-coated slides and incubated at 37 °C for 30 min. The plates were then rinsed with PBS for 11 min and allowed to dry completely. Then, 20 µl of commercial secondary anti-dog IgG antibody produced in rabbits and conjugated with fluorescein isothiocyanate (AffiniPure Rabbit Anti-Dog IgG, Jackson ImmunoResearch Laboratories Inc., Suffolk, United Kingdom) was added to each well at a 1/100 dilution in PBS at pH 7.2, and Evans blue was used as a control (at a final concentration of 1/1000). The slides were then incubated at 37 °C for 30 min, followed by repeated rinsing and drying steps. The slides were air-dried at room temperature in darkness and mounted with commercially available buffered glycerine.

Slides were examined via an Olympus BH-2® epifluorescence microscope (Olympus Imaging America Inc., Center Valley, Pennsylvania, United States) with a blue filter and ×400 objective. The exact same IFAT protocol and epifluorescence microscope were employed in both the initial and repeated serological tests.

The antibody titre for each sample was determined by identifying the highest dilution at which specific fluorescence was observed. In this study, the endpoint dilution was set at 1/6400, and the positivity cut-off was established at ≥1/100. To minimise the potential for error, the original serological status of the samples was blinded, and the plasma samples were re-examined by three experienced IFAT evaluators.

### Data analysis

The initial and subsequent serological analysis data were organised in a data file. The quantitative titre and qualitative outcome (positive: titre ≥1/100; negative: titre <100) from both examinations were recorded. Furthermore, each sample result was classified via a binary system with ‘yes’ and ‘no’ categories on the basis of several criteria: quantitative agreement (concordance between the former and posterior quantitative titres) and qualitative agreement (concordance between the former and posterior qualitative outcomes). Owing to the observer-dependent nature of the technique and the current evidence on the diagnosis of this infection using serological methods, a discrepancy of one titre immediately higher or lower (onefold difference) between the former and current determination was considered acceptable, whereas differences greater than this value were deemed unacceptable [10, 32–34]. This criterion has been referred to as an acceptable discrepancy.

### Statistical analysis

**Fig. 1:**
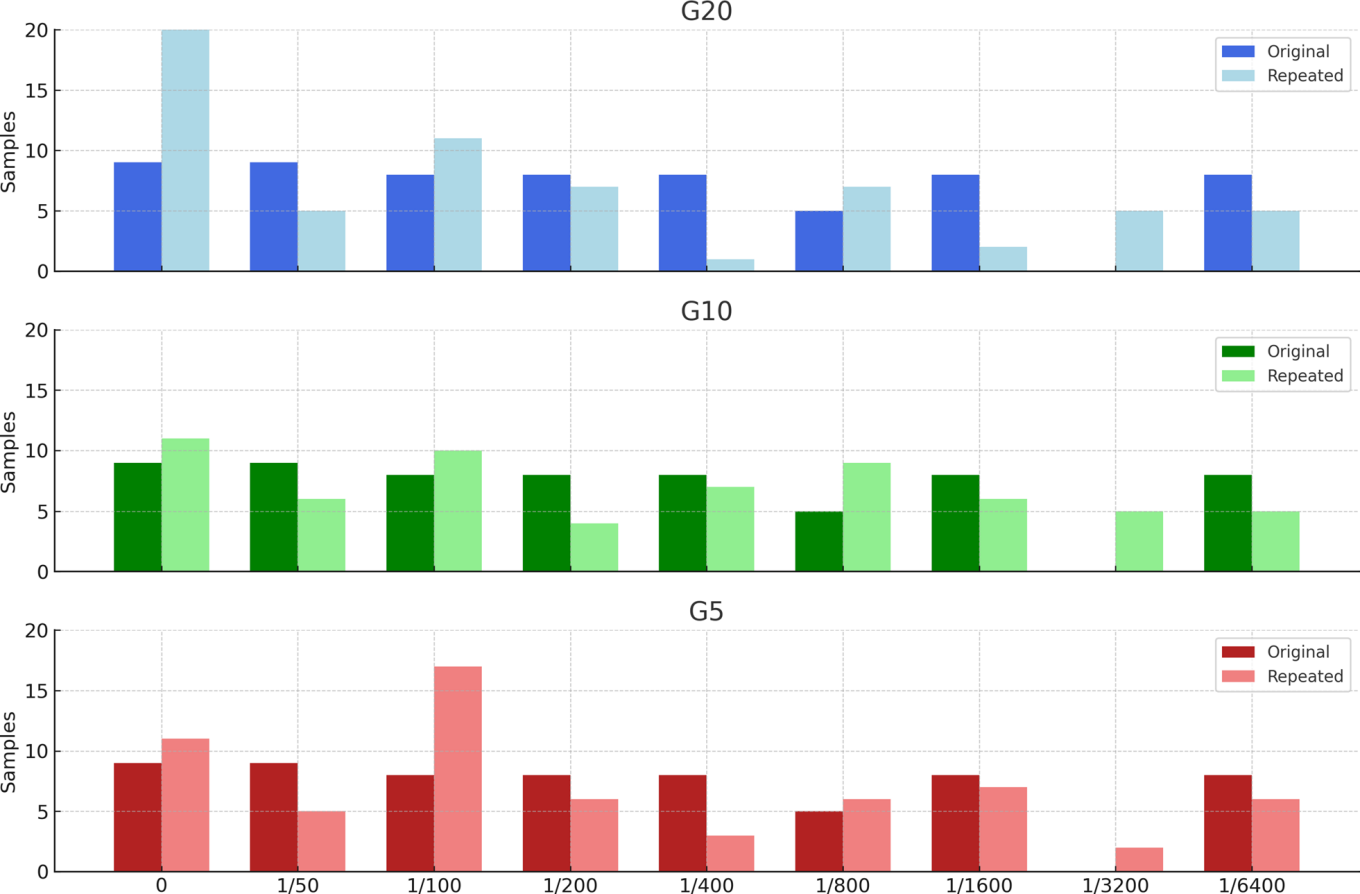
Quantitative results of the original and repeated IFAT determinations. The number of samples is represented on the vertical axis, and the IFAT titre is represented on the horizontal axis. Abbreviations: G20, samples from 20 years; G10, samples from 10 years; G5, samples from 5 years; Original, first IFAT determination performed prior to frozen storage. The second IFAT determination performed from July to November 2024

The results were statistically analysed via IBM SPSS Statistics software v.29.0.2.0 (IBM, Armonk, NY, USA). A descriptive analysis was performed on the numeric (mean and standard deviation; median and range) and categoric (frequency and percentage) variables for the total number of samples and for each individual group. For each of them, significant differences between the previous and current IFAT outcomes were analysed via different statistical analyses. As the Shapiro‒Wilk test indicated a lack of normal distribution in the quantitative titres, a Wilcoxon signed-rank test and Spearman’s correlation analysis were used to assess the differences between the former and current quantitative titres. To evaluate the qualitative outcome (positive or negative), the McNemar test and Cohen’s kappa test were employed to assess the degree of agreement between the original IFAT outcome and the repeated outcome. According to established conventions, *κ* values of 0–0.2 are designated as slight, 0.2–0.4 as fair, 0.4–0.6 as moderate, 0.6–0.8 as substantial and 0.8–1.0 as almost perfect agreement. The quantitative agreement, qualitative agreement and acceptable discrepancy of the dichotomous variables were evaluated using the chi-square test or Fisher’s exact test, as appropriate. A statistically significant difference was set at *P* < 0.05. Figure 1 was generated using Python (v3.11) and Matplotlib (v3.8.0) on the basis of quantitative IFAT titre data from original and repeated determinations across three age groups (G20, G10 and G5).

## Results

The global percentage of qualitative agreement (positive or negative) revealed that 92.1% (*n* = 174/189) of the samples were consistent over time. Specifically, the qualitative outcome showed a greater degree of coincidence in the group of samples from 10 years prior (G10), at 98.4% (*n* = 62/63). Both G20 and G5 presented the same percentage of matches at 88.9% (*n* = 56/63) (*χ*^2^ = 5.2138; *df* = 2; *P* = 0.0738).

In the course of the analysis of the 189 samples, 135 (71.4%) were positive (≥1/100) and 54 (28.6%) were negative at the initial measurement, regardless of when they were measured. Over time, these percentages shifted in a non-significant manner, with the number of positive samples decreasing to 131 (69.3%) and the number of negative samples increasing to 58 (30.7%) (*P* = 0.2850; *κ* = 0.8225). These data were also analysed by groups. At the time of the initial measurement, each of the three groups consisted of 63 samples, of which 45 (71.4%) were positive and 18 (28.6%) were negative. In the posterior measurement, these percentages varied depending on the group. The samples aged 20 years presented the highest percentage of negative samples in the second analysis (39.7%; *n* = 25; *P* = 0.0082; *κ* = 0.7562), followed by the samples aged 10 years (27%; *n* = 17; *P* = 0.3173; *κ* = 0.9605) and finally, the 5-year-old samples (25.4%; *n* = 16; *P* = 0.4142; *κ* = 0.7586). The qualitative results of the original and repeated IFAT determinations are presented in Table 1. The results presented correspond to different samples that were originally evaluated and subsequently re-evaluated after 20 years (G20), 10 years (G10) and 5 years (G5). These samples are independent and represent separate measurements conducted at the specified time intervals.

**Table 1:** Qualitative results of the original and repeated IFAT determinations

Qualitative determination		Negative (<1/100)	Positive (≥1/100)	Total
Original	Total	54 (28.6%)	134 (71.4%)	189 (100%)
	G20	18 (28.6%)	45 (71.4%)	63
	G10	18 (28.6%)	45 (71.4%)	63
	G5	18 (28.6%)	45 (71.4%)	63
Repeated	Total	58 (30.7%)	131 (69.3%)	189 (100%)
	G20	25 (39.7%)	38 (60.3%)	63
	G10	17 (27%)	46 (73.0%)	63
	G5	16 (25.4%)	47 (74.6%)	63

G20, samples from 20 years; G10, samples from 10 years; G5, samples from 5 years; Negative (<100), number of samples with an IFAT titre <1/100; Original, first IFAT determination prior to frozen storage; Positive (≥100), number of samples with an IFAT titre ≥1/100; Repeated, second IFAT determination performed from July to November 2024

Upon analysis of all samples collectively, the quantitative antibody titres had a median titre of 1/200 (range: 0–1/6400) in the initial measurement and a median titre of 1/100 (range: 0–1/6400) in the current measurement. The difference between these two quantitative results was found to be significant (*Z* = −706; *P* = 0.0079). Further analysis by the time of initial measurement revealed that the median titre of samples from 20 years prior (G20) and 5 years prior (G5) decreased from 1/200 to 1/100. However, the discrepancy in the quantitative titre between the two time periods was statistically significant only for the G20 samples (*Z* = −243; *P* < 0.001 and *Z* = −57.5; *P* = 0.2656, respectively). In contrast, samples measured 10 years ago (G10) exhibited an increase from a median titre of 1/200 to 1/400, although the quantitative difference between both analyses was deemed non-significant (*Z* = 12; *P* = 0.7895).

A strong positive correlation was observed between the quantitative measurements of the past and present across all three groups: G20 (*rs* = 0.9335, *P* < 0.001), G10 (*rs* = 0.9546, *P* < 0.001) and G5 (*rs* = 0.9240, *P* < 0.001).

The percentage of samples that exactly matched the specific quantitative titre was evaluated. A total of 48.7% (*n* = 92/189) of the samples analysed had the same exact titre in both examinations. Specifically, 42.9% (*n* = 27/63) of the 20-year-old samples, 55.6% (*n* = 35/63) of the ten-year-old samples and 47.6% (*n* = 30/63) of the 5-year-old samples matched (*χ*^2^ = 2.0755; *df* = 2; *P* = 0.3542). The quantitative results of the original and repeated IFAT determinations can be found in Table 2 and Fig. 1.

**Table 2:** Quantitative results of the original and repeated IFAT determinations

Quantitative determination	Original				Repeated			
IFAT titre	Total	G20	G10	G5	Total	G20	G10	G5
0	27 (14.3%)	9	9	9	42 (22.2%)	20	11	11
1/50	27 (14.3%)	9	9	9	16 (8.5%)	5	6	5
1/100	24 (12.7%)	8	8	8	38 (20.1%)	11	10	17
1/200	24 (12.7%)	8	8	8	17 (9%)	7	4	6
1/400	24 (12.7%)	8	8	8	11 (5.8%)	1	7	3
1/800	15 (7.9%)	5	5	5	22 (11.6%)	7	9	6
1/1600	24 (12.7%)	8	8	8	15 (7.9%)	2	6	7
1/3200	0 (0%)	0	0	0	12 (6.4%)	5	5	2
1/6400	24 (12.7%)	8	8	8	16 (8.5%)	5	5	6
Total	189 (100%)	63 (33.3%)	63 (33.3%)	63 (33.3%)	189 (100%)	63 (33.3%)	63 (33.3%)	63 (33.3%)

G20, samples from 20 years; G10, samples from 10 years; G5, samples from 5 years; Original, first IFAT determination performed prior to frozen storage; Second IFAT determination performed from July to November 2024

The percentage of acceptable discrepancy was 86.2% (*n* = 163/189). Specifically, an acceptable discrepancy was found in 82.5% (*n* = 52/63) of the observed titres in G20 and G5 and 93.7% in G10 (*n* = 59/63) (*χ*^2^ = 4.3705; *df* = 2; *P* = 0.1125).

## Discussion

In the present study, three sets of plasma samples whose serological status against *L. infantum* was previously determined using IFATs and stored at −20 °C for 5, 10 and 20 years were reassessed. Considerable qualitative agreement (92.1%) was found between the original and repeated serological results, suggesting that IgG antibody determination by IFAT demonstrates a reasonable degree of reproducibility following extended sample storage at freezing temperatures.

This finding is consistent with that of a recent study from Karagkouni et al. [19], which assessed the stability of IgG antibodies against *Ehrlichia canis* in samples stored frozen for long periods of time via the same diagnostic technique. The study reported a 90.9% agreement rate. Consequently, it could be hypothesised that the stability of canine IgGs against different pathogens at a temperature of −20 °C is high and that this stability might not be limited solely to anti-*Leishmania*-specific IgG antibodies.

With the passage of time, a marked increase in the proportion of samples yielding negative results was observed in the oldest samples (20 years). In this initial group (G20), the percentage of quantitative agreement is the lowest of the three, although not significantly so. One potential explanation for this phenomenon could be the onset of antibody degradation during this period of storage. The absence of an increase in the percentage of negative samples in the groups stored for 5 and 10 years suggests that the potential degradation of IgG against this agent begins after 10 years. Further studies using samples stored for periods ranging from 10 to 20 years would be advisable to assess whether gradual degradation of these molecules occurs and to what extent it may affect the various serological titres. In the study by Dard et al. [26], the stability of human anti-*Toxoplasma* IgGs in samples frozen at −20 °C for 10 years was analysed, and it was also found that the variability in the antibody titre over time did not follow a consistent negative trend and that IgGs were stable under these conditions for at least that time period.

The findings of the present study align with those of several others, supporting the use of freezing at −20 °C as an effective method for preserving immunoglobulin samples for a period of years [35–37]. Although these previous studies have been conducted in human samples, some studies have evaluated human and canine anti-*Leishmania*-IgGs in conjunction, obtaining analogous results in both species via the direct agglutination test [20, 21]. Therefore, it could be presumed that the stability of canine IgGs may be similar to that observed in humans, although it should be taken into consideration that they might not be structurally identical [37, 38]. While IFAT is a semiquantitative technique that may limit the detection of subtle antibody degradation over time, it remains the reference standard for the serological diagnosis of canine leishmaniosis. Future studies incorporating complementary quantitative methodologies are warranted to further characterise antibody stability in long-term stored samples. In this sense, Marteles et al. [39] recently evaluated the stability of IgG antibodies against *Leishmania infantum* in canine serum stored at −20 and −80 °C for 2.5 years via direct quantification via ELISA. These results, which were consistent with our own findings, demonstrated high antibody stability, thus supporting the use of frozen samples in retrospective serological studies despite methodological differences.

IFAT is a highly recommended technique for the serological diagnosis of *L. infantum* exposure [1, 4–7]. However, it is subject to intrinsic variability, which is partly attributable to its observer-dependent interpretation [4, 10, 32, 33]. A limitation of this study is that it was not possible to evaluate the potential influence owing to the individual interpretation of the observer. In the context of long-term studies of this nature, it is inevitable that IFAT interpreters have changed over time. Notably, however, all the interpreters had undergone rigorous training by their predecessors. Nevertheless, it is crucial to emphasise that even if the bias is reduced, the same interpreter may obtain slightly divergent results for the same sample at different times. As discussed by other authors [10, 32, 33], this is an inherent limitation of the technique, emphasising the importance of interpreting the results while considering this circumstance. A decrease in the IFAT titre would be considered significant if there was more than a twofold dilution difference between the first and subsequent samples [34]. The present study therefore evaluated the variable acceptable discrepancy, which obtained a high percentage across all groups.

When using serological diagnostic methods, such as IFATs, it is important to consider the possibility of cross-reactions between different agents. In the context of the serological diagnosis of *Leishmania* spp., cross-reactivity with *Trypanosoma cruzi* has been documented [40, 41], although this agent is not present in the geographical area where the animals in this study originated. Cross-reactions with other pathogens, such as *Ehrlichia canis*, *Babesia canis* and *Toxoplasma gondii*, have not been found to be relevant according to the current literature [40, 42–44].

Considering the aforementioned points, it can be hypothesised that a significant proportion of the observed variability between the initial and subsequent IFAT results is attributable to the inherent variability of the technique itself. Therefore, the results should be interpreted in light of this inherent limitation. However, it is noteworthy that the qualitative assessment of frozen samples has a high degree of reproducibility. This is a particularly relevant consideration within the clinical context, where samples can be examined for *L. infantum* exposure retrospectively and for research purposes, where sampling and subsequent analysis frequently occur at disparate temporal points.

## Conclusions

The findings of this investigation demonstrate that IgG antibody determination against *L. infantum* using IFAT has a high degree of reproducibility, even following prolonged freezing at −20 °C for up to 20 years. These observations suggest that long-term storage of plasma samples does not substantially affect the qualitative accuracy of serological tests, supporting the utilisation of this preservation method for clinical and research purposes. Despite some observed variability, the overall stability of canine IgGs at −20 °C aligns with previous studies on both human and canine samples. The high qualitative agreement between the initial and repeated results underscores the reliability of the IFAT for serological assessments over time. These findings emphasise the utility of frozen plasma storage for maintaining the integrity of serological samples, particularly in longitudinal studies and large-scale epidemiological research.

## Data Availability

Data supporting the main conclusions of this study are included in the manuscript.

## Abbreviations

CanL: Canine leishmaniosis
IFAT: Indirect immunofluorescence antibody test
IgG: Immunoglobulin G
PBS: Phosphate-buffered saline
